# COVID-19 Vaccine Framing and Acceptance Among Adults Who Are Vaccine Hesitant

**DOI:** 10.1001/jamanetworkopen.2026.4114

**Published:** 2026-03-31

**Authors:** Parthasarathy Krishnamurthy, Ye Hu

**Affiliations:** 1 C. T. Bauer College of Business, University of Houston, Houston, Texas

## Abstract

**Importance:**

Autonomy concerns represent one of many contributors to vaccine hesitancy, yet public health messaging often emphasizes government compliance. It is important to study whether alternative message framing is associated with stated vaccine acceptance among hesitant individuals.

**Objective:**

To evaluate whether framing vaccines as freedom enhancing is associated with higher vaccine acceptance among vaccine hesitant adults.

**Design, Setting, and Participants:**

This cross-sectional online discrete choice experiment was conducted from May 1 to 4, 2024. Each respondent completed 12 choice tasks comparing 2 vaccine options and a no-vaccine option, with vaccines described by 6 attributes, including infection prevention and severe disease protection efficacy, minor and major adverse effects, duration of protection, and additional reason to take (message framing).

**Exposures:**

Three levels for message framing, including government compliance (reference), personal freedom, and protect others.

**Main Outcomes and Measures:**

Hesitancy was measured using vaccine concerns and the Vaccine Adverse Belief Index (VABI). Main outcomes were vaccine preference shifts and estimated vaccine uptake associated with message framing variations examined using hierarchical bayesian analysis, with vaccine concerns and VABI serving as second-level factors.

**Results:**

A total of 907 adults (mean [SD] age, 69.5 [11.0] years; 454 female [50.3%]) participated in the study. Among respondents with high vaccine concern, freedom framing was associated with a preference shift of 33.8 percentage points (95% credible interval [CrI], 21.5-45.8 percentage points) and an increase in uptake of 6.3 percentage points (95% CrI, 2.9-11.5 percentage points), assuming the most favorable vaccine profile (highest efficacy, lowest adverse effects, longest duration). Among respondents with a high VABI, freedom framing was associated with a preference shift of 27.7 percentage points (95% CrI, 14.5-40.5 percentage points) and an increase in uptake of 4.6 percentage points (95% CrI, 1.8-8.8), respectively. Respondents with low vaccine concern or VABI showed no significant preference shifts associated with framing. In contrast, protect-others framing was associated with positive preference shifts and uptake, with no evidence that these associations differed by vaccine hesitancy.

**Conclusions and Relevance:**

This cross-sectional study found that compared with government recommendation framing, freedom framing was selectively associated with higher vaccine acceptance among adults who were vaccine hesitant. Protect-others framing was associated with higher vaccine acceptance regardless of hesitancy level. Absolute gains in acceptance were modest. These findings highlight how autonomy-consistent framing may influence stated vaccine preferences among hesitant adults.

## Key Points

**Question:**

Is freedom framing associated with COVID-19 vaccine acceptance among individuals who have concerns?

**Findings:**

In this cross-sectional study using a discrete choice experiment among 907 adults in the US, freedom framing compared with government recommendation framing was associated with substantial preference shifts among respondents expressing vaccine concerns or endorsing adverse beliefs about COVID-19 vaccines, with no such association among respondents with low vaccine concerns.

**Meaning:**

These findings suggest that autonomy-consistent framing may influence stated vaccine preferences among vaccine hesitant adults, with implications for both targeted and broadly applicable communication strategies.

## Teaser

This cross-sectional study evaluates whether COVID-19 vaccine message framing emphasizing personal autonomy is associated with vaccine acceptance among adults who are vaccine hesitant.

## Introduction

Vaccination campaigns remain among the most effective public health interventions, preventing an estimated 154 million deaths globally over the past 5 decades.^1^ In the US alone, childhood vaccines have averted more than 508 million illnesses and more than 1 million deaths since 1994.^2^ Despite these successes, coverage is plateauing or declining across multiple vaccines, with recent influenza (48.4%) and COVID-19 booster (22.5%) uptake falling below public health targets.^3,4^ Localized clusters of unvaccinated individuals continue to be at risk for outbreaks of vaccine-preventable diseases, such as measles.^5^ These patterns highlight the persistence of vaccine hesitancy despite broad availability and strong safety profiles.

Vaccine hesitancy, defined as the delay or refusal to accept vaccines despite availability,^6,7,8^ is a complex phenomenon with multiple determinants. Within Moral Foundations Theory, the liberty or oppression foundation has been associated with vaccine hesitancy in prior work,^9^ situating autonomy-related considerations as 1 dimension along which hesitant and nonhesitant individuals may differ.

Prevailing vaccination messages frequently emphasize institutional authority and endorsement, framing vaccination as a government-recommended or expert-driven action.^10^ Messages designed to promote vaccination can be perceived as constraining personal choice, especially in contexts in which autonomy concerns are salient.^6,11^ If vaccine acceptance reflects preferences constructed in response to decision contexts rather than a fixed trait,^12,13^ communication approaches that align with autonomy may be associated with higher acceptance.

As an alternative to authority-based messaging, vaccination could be framed in ways that emphasize personal freedom, ie, freedom framing. Freedom framing involves describing vaccination as a means of preserving or restoring personal functional autonomy, including freedom from infection, severe illness, and lifestyle restrictions that illness imposes, rather than as an act of compliance or altruism. Unlike traditional autonomy restoration approaches that append choice-affirming statements to standard messages,^14,15^ freedom framing centers autonomy as part of the rationale for vaccination.

This study examines whether associations between vaccine framing and vaccine acceptance differ across individuals with varying levels of vaccine hesitancy. Research on value congruence has suggested that individuals overweight decision attributes aligned with their core concerns.^16^ Accordingly, we hypothesized that freedom framing would be positively associated with vaccine acceptance among individuals with higher levels of vaccine hesitancy than among those with lower hesitancy.

Vaccine communication also frequently uses protect-others framing, which emphasizes communal responsibility by highlighting the benefits of vaccination for reducing transmission risk to others. However, this rationale does not center around personal autonomy concerns. Accordingly, we do not advance a specific hypothesis about whether vaccine hesitancy moderates the association between protect-others framing and vaccine acceptance. We examined these associations between vaccine message framing and stated vaccine acceptance using a discrete choice experiment (DCE) in which vaccine attributes, including framing, are systematically varied across hypothetical vaccination decisions.

## Methods

### Study Design and Respondents

This cross-sectional study was conducted using a web-based DCE, consistent with established good research practices for conjoint analysis,^17,18^ to examine how vaccine attributes and framing are associated with preferences among adults in the US. The study was approved by the University of Houston Institutional Review Board. The survey was administered using the Qualtrics experience management platform, which manages informed consent and links respondents to the Conjointly survey research platform (Analytics Simplified Pty Ltd) for the DCE component via pass-through identifiers. The study followed the Strengthening the Reporting of Observational Studies in Epidemiology (STROBE) reporting guideline for cross-sectional studies.^19^

The DCE survey development details are provided in eAppendix 1 in Supplement 1. Data were collected from May 1 to 4, 2024. Respondents were recruited through Prime Panels (CloudResearch), an opt-in, nonprobability online panel, with targeted equal representation (33%) of Democratic, Independent, and Republican affiliations. Based on the Orme rule of thumb^20,21^ (500L*^max^*/*ta*, where L*^max^* is the maximum attribute levels, *t* is the number of choice tasks, and *a* is the number of alternatives per task), our design required a minimum of 84 respondents. Although the DCE platform recommended 200 respondents, we targeted approximately 1000 to allow for robust modeling of preference heterogeneity. The final sample included respondents who passed both attention checks and automated quality control procedures (eAppendix 2 in Supplement 1).

### Survey Structure and DCE Design

After providing informed consent, respondents were redirected to the survey research platform, which implemented the DCE. Each respondent completed 12 randomized choice sets, each presenting 2 hypothetical vaccines described by 6 attributes, plus an opt-out option (“I will not choose either vaccine”). The attributes were derived primarily from a review of attributes in DCEs, which revealed primarily 3 outcome-related attribute types: effectiveness, risk, and duration of protection.^22^ Our DCE design used 6 attributes: infection prevention efficacy, severe disease protection efficacy, chance of minor adverse effects, chance of major adverse effects, duration of protection, and the additional reason for taking the vaccine (message framing). The complete list of attributes, attribute levels, and respondent-facing wording is provided in Table 1.

**Table 1.** Vaccine Attributes and Levels in the Discrete Choice Experiment

Attribute^a^	Details	Levels
Efficacy in preventing infection (infection prevention efficacy)	“This describes how effective the vaccine is in preventing you from getting the infection.”	50% 65% 80% 95%
Efficacy in preventing hospitalization and death if infected (severe disease protection efficacy)	“This describes how effective the vaccine is in preventing the most severe consequences of infection such as hospitalization or death.”	50% 65% 80% 95%
Chance of minor side effects (minor adverse effects)	“This describes the chance of getting minor side effects such as short-term fever or body ache that goes away in a couple of days and is easily treated with over-the-counter medications such as [acetaminophen].”	50% 70% 90%
Chance of major adverse effects (severe adverse effects)	“Severe side effects that require hospitalization or long-term treatment are very rare. This describes how many people typically experience severe side effects.”	1 in 100 000 1 in 500 000 1 in 1 000 000
Duration of protection (duration of protection)	“How long will the vaccine protect you from (infection and severe disease as described above)?”	6 mo 5 y 10 y
Additional reason for taking (additional reason for taking)	“The primary reason why it is a good idea to take the vaccine:”	Comply with government recommendations Help prevent spread of disease Personal freedom to do what you want to do

^a^ Attribute descriptions shown outside parentheses reflect the exact wording presented to respondents. Labels shown within parentheses are terms used throughout the article and figures for clarity of presentation.

The survey research platform generated a fractional factorial choice design optimized for balance and minimal overlap and randomly assigned each respondent to a block. Within each block, the order of choice tasks and the presentation order of alternatives were randomized to minimize order bias.

Upon completion of the DCE, respondents were automatically redirected back to the experience management platform to answer demographic and psychological measures. A screenshot of a typical choice set is provided in eFigure 1 in Supplement 1.

The second section of the survey collected respondents’ COVID-19 vaccination status, demographics (sex, self-reported race and ethnicity to characterize the sample [White or other (African American, Asian, Hispanic, Native American or Pacific Islander, multiethnic, prefer not to answer, and other)], age, income, education, and living arrangement), and attention check items. Race and ethnicity were used to characterize the sample, and the other category was combined due to small numbers of respondents. Vaccine hesitancy was operationalized using 2 complementary psychological measures: vaccine concerns and vaccine adverse beliefs.

Vaccine concerns were assessed using validated items from the Vaccination Attitudes Examination scale,^23^ which reflects general attitudes toward vaccination. Vaccine adverse beliefs were assessed through the Vaccine Adverse Beliefs Index (VABI),^24,25,26^ which measures agreement with statements based on myths about COVID-19 vaccines identified by the Mayo Clinic and Cleveland Clinic,^24,25^ as well as opinion-based statements reflecting vaccine development, safety, and institutional trustworthiness. Both vaccine concern and VABI measurements are detailed in eAppendix 3 in Supplement 1. The complete survey, including respondent-facing instructions, question wording, and response options, is provided in eAppendix 4 in Supplement 1.

### Statistical Analysis

We analyzed the DCE data using a random parameters logit model with bayesian estimation using the PROC BCHOICE procedure in SAS, version 9.4 (SAS Institute Inc).^27,28^ Key features of our analysis included incorporation of a dummy variable for the opt-out option^29^ and effect coding for attribute levels^30,31^ (additional model details provided in eAppendix 3 in Supplement 1). Conditional relative importance was calculated by dividing each respondent’s attribute-specific preference range by the sum of ranges across all attributes, scaled to 100. Preference weights were extracted from posterior draws as attribute-level part-worth utilities; omitted levels were recovered as the negative sum of estimated levels within each attribute, and posterior means and 95% credible intervals (CrIs) of these derived quantities are shown.

Our primary analysis used a 2-level hierarchical model^32^ to examine whether freedom framing is associated with higher vaccine preference among respondents with heightened vaccine hesitancy, as indicated by vaccine concern and VABI. The first level used hierarchical bayesian multinomial logit regression with bayesian pooling^33^ to identify individual-level coefficients, which then served as dependent variables in a linear regression with vaccine concerns as the independent variable. Thus, we had 2 different models, each based on a different correlate of vaccine hesitancy, vaccine concern and VABI, to model how hesitancy moderates preference weights. We conducted the second-level analyses using both a binary indicator of vaccine concern and VABI (based on a median split) and the composite continuous scores thereof. All key findings were robust when using the continuous versions. In the interest of expositional and graphical simplicity, we present the results from the binary indicator. Primary inference focused on the prespecified directional contrast comparing freedom framing with government recommendation framing and its association with vaccine acceptance across levels of vaccine hesitancy. Analyses of contrasts involving protect-others framing were exploratory.

For each of the 2 models, we characterized the estimated size using 2 complementary variable size measures calculated from the hierarchical bayesian posterior draws to quantify the practical significance of estimated size of government vs freedom framing. The 2 estimated size measures were (1) pairwise preference shifts and (2) absolute uptake changes, quantified using framing association from posterior draws using a favorable vaccine profile (95% infection prevention, 95% severe disease prevention, 50% minor adverse effects, 1:100 000 major adverse effects, 10-year duration). Pairwise preference shifts used tanh(ΔU/2) × 100 (where tanh denotes the hyperbolic tangent function and ΔU represents the difference in estimated utilities between the 2 framing conditions) to convert utility differences between freedom and government framing into percentage point deviations from 50% baseline preference, ignoring opt-out behavior. We defined the probability of selecting any vaccine vs opting out as P (any vaccine) = 2*e^Uvaccine^*/(2*e^Uvaccine^* + *e^Uopt-out^*), where *Uvaccine* is the utility of the benchmark vaccine profile and *Uopt-out* is the utility of opting out. This formulation used neutral opt-out baselines to isolate how framing is associated with acceptance. Both vaccine alternatives were specified as identical profiles on all attributes except framing so that uptake reflects the probability of choosing any vaccine vs opting out rather than differences between vaccine profiles, consistent with standard DCE simulation practice.^30,34,35^ The shift and uptake estimates associated with framing were computed across 4 vaccine profile scenarios (best, medium, mixed, and worst) to assess sensitivity. Although we did not advance specific anticipations regarding protect-others framing, we computed shift and lift estimates for this comparison between government recommendation and protect-others framing for completeness. Missing demographic data were minimal (<1% for most variables) and were not imputed; analyses used all available data. A 95% CrI excluding 0 was considered indicative of association.

## Results

### Respondents

Of 1465 individuals who consented and accessed the study, 907 (61.9%; 454 female [50.3%] and 448 male [49.7%]) completed all components, passed automated quality control and attention checks, and were included in the analysis. The respondents were balanced for political affiliation (331 Democrat [36.5%], 274 Independent [30.2%], and 301 Republican [33.2%]). Compared with the general US population,^36,37^ the sample had a slightly higher educational attainment (494 completed college [54.5%]), was older on average (mean [SD] age, 69.5 [11.0] years), and was predominantly White (803 identifying as White [89.1%] compared with 98 identifying as another [10.9%] race and ethnicity). Most respondents had received at least 1 dose of the COVID-19 vaccine (783 [86.3%]) and the influenza vaccine (642 [71.0%]) and scored near the midpoint of the vaccine concern measure (mean [SD], 3.2 [1.1] on a scale of 0-6, with higher scores indicating more concerns). High and low groups for vaccine concern and VABI were created through a split of their median scores (3.0 [IQR, 2.3-3.8] and 2.2 [IQR, 1.5-3.1], respectively). Additional sample details are provided in Table 2.

**Table 2.** Respondent Characteristics

Measure	Respondents (N = 907), No. (%)
Age, y	
Mean (SD) [range]	69.5 (11.0) [18.0-100.0]
Missing, No.	2
Sex	
Female	454 (50.3)
Male	448 (49.7)
Missing, No.	5
Race and ethnicity	
White	803 (89.1)
Other^a^	98 (10.9)
Missing, No.	6
Education	
Completed college and above	494 (54.5)
Up to some college	413 (45.5)
Income, $1000	
Mean (SD) [range]	67.9 (46.9) [0-200.0]
Missing, No.	4
Political affiliation	
Democrat	331 (36.5)
Independent	274 (30.2)
Republican	301 (33.2)
Missing, No.	1
COVID-19 vaccination status	
No vaccine	124 (13.7)
≥1 Dose	783 (86.3)
Composite COVID-19 direct impact score^b^	
Mean (SD) [range]	1.6 (1.3) [0.0-5.0]
Missing	0
Vaccine concerns (composite measure)^c^	
Mean (SD) [range]	3.2 (1.1) [1.0-6.0]
Missing	0
Vaccine concerns (median split)^d^	
Low	463 (51.0)
High	444 (49.0)
Vaccine Adverse Belief Index (composite measure)^e^	
Mean (SD) [range]	2.5 (1.1) [1.0-6.0]
Missing	0
Vaccine Adverse Belief Index (median split)^d^	
Low	457 (50.4)
High	450 (49.6)

^a^ Included African American, Asian, Hispanic, Native American or Pacific Islander, multiracial, or other.

^b^ Scale of 0 to 5, with higher values indicating greater direct impact of COVID-19.

^c^ Scale of 1 to 6, with higher values indicating greater vaccine concern.

^d^ Median splits for vaccine concerns and Vaccine Adverse Belief Index were defined as scores at or below the median classified as low and scores above the median classified as high.

^e^ Scale of 1 to 6, with higher values indicating stronger endorsement of adverse vaccine beliefs.

### Overall Preferences for Vaccine Attributes

**Figure 1.**
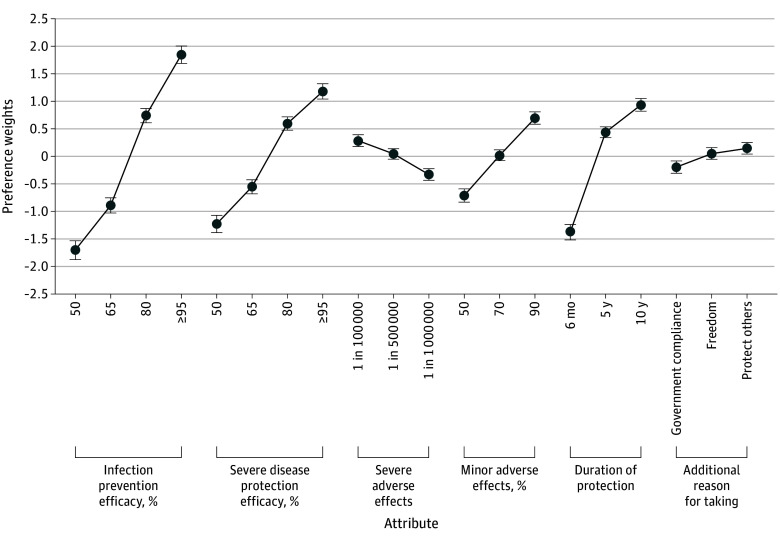
Line Graph of Estimates of Preferences for Vaccine Attributes and Levels Points indicate the mean part-worth utilities; error bars, the 95% credible intervals from a hierarchical Bayes model. Higher values indicate greater preference. Utility differences are comparable across attributes, eg, the drop when infection prevention declines from 95% to 80% is similar to the gain from extending protection from 6 months to 10 years.

The preference weights for the 6 attributes and their levels are shown in Figure 1. The ordering of preference weights within each attribute was consistent with expectations, with more favorable levels corresponding to higher preference weights (eg, higher infection prevention efficacy was preferred over lower infection prevention efficacy).

The relative importance of attributes, derived from the estimated preference weights from the DCE, are presented in eFigure 2 in Supplement 1, and the corresponding regression results are provided in eTable 1 in Supplement 1. All 6 attributes affected vaccine preferences, though their importance varied. Infection prevention efficacy was the most important attribute (mean, 33.4%; 95% CrI, 31.2%-35.6%), followed by duration of protection (mean, 21.7%; 95% CrI, 19.8%-23.7%) and severe disease protection (mean, 22.7%; 95% CrI, 20.8%-24.6%), which did not differ significantly from each other. Severe adverse effects (mean, 13.2%; 95% CrI, 11.4%-15.0%) were less important than the preceding attributes but more important than minor adverse effects (mean, 5.7%; 95% CrI, 4.1%-7.4%) and framing (mean, 3.2%; 95% CrI, 1.6%-4.9%), which were not significantly different from each other. Although the framing attribute had a low relative importance overall, its relevance became clear when heterogeneity in vaccine concern and VABI was taken into consideration, as shown in the second-level analyses next.

### Association Between Vaccine Hesitancy and Vaccine Preferences

**Figure 2.**
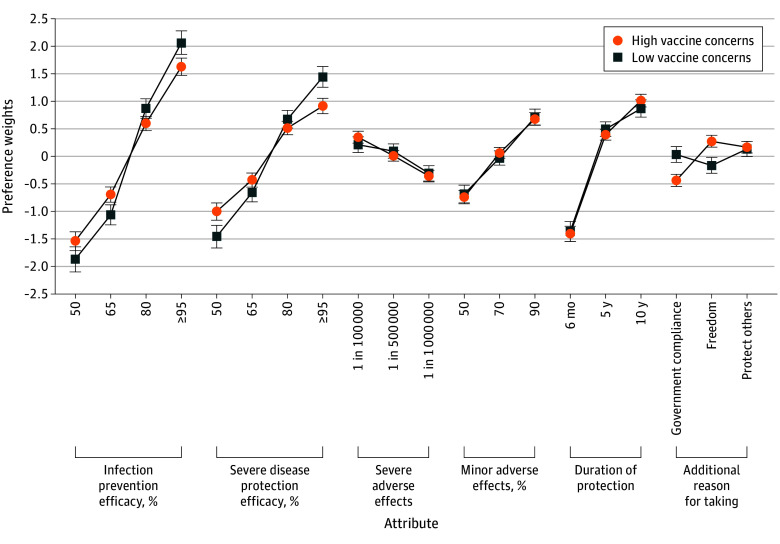
Line Graphs of Vaccine Attribute Preference by Vaccine Concerns Markers indicate mean preference weights; error bars, the 95% credible intervals (CrIs). Attribute levels were effects coded. Estimates for generating this figure are reported in eTable 2 in Supplement 1. For each posterior draw, group-specific utilities were reconstructed by combining the pooled-level coefficient with the corresponding effects-coded group interaction term. Within each draw, the utility for the omitted level of each attribute was recovered as the negative sum of the remaining levels so that utilities sum to 0 within the attribute. Plotted estimates are posterior means across draws; CrIs correspond to the 2.5th and 97.5th percentiles of the draw-level utilities.

**Figure 3.**
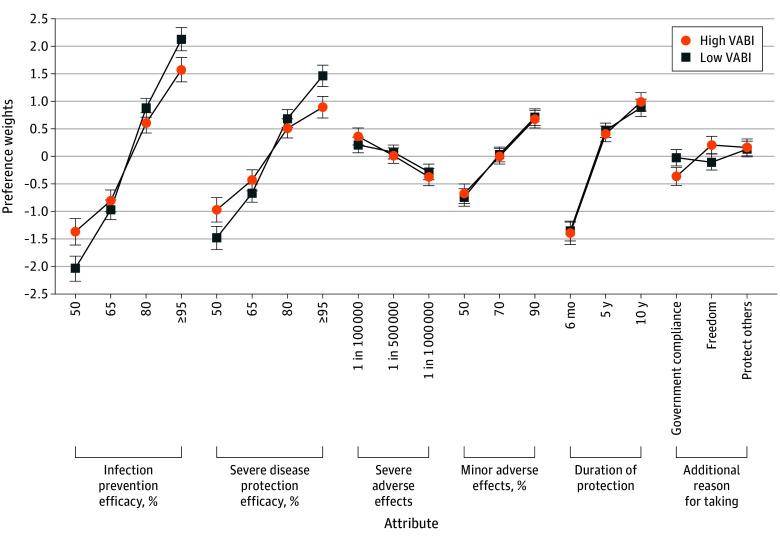
Line Graphs of Vaccine Attribute Preference by Vaccine Adverse Belief Index (VABI) Markers indicate mean preference weights; error bars, 95% credible intervals (CrIs) by VABI group. Attribute levels were effects coded. Estimates for generating this figure are reported in eTable 3 in Supplement 1. For each posterior draw, group-specific utilities were reconstructed by combining the pooled-level coefficient with the corresponding effects-coded group interaction term. Within each draw, the utility for the omitted level of each attribute was recovered as the negative sum of the remaining levels so that utilities sum to 0 within the attribute. Plotted estimates are posterior means across draws; CrIs correspond to the 2.5th and 97.5th percentiles of the draw-level utilities.

To examine whether vaccine preferences varied by level of vaccine hesitancy, we used a 2-level mixed-effects multinomial logit regression using hierarchical Bayes estimates. At the first level, vaccine choices were modeled using 6 attributes. The resulting individual-level coefficients (summarized in Figure 1) were then used in a second-level linear regression with the binary version of the 2 indicators of hesitancy, vaccine concern and VABI, as the independent variable. The preferences for attributes by level and by moderator vaccine concern and VABI are presented in Figure 2 and Figure 3 (generated from estimates reported in eTables 2 and 3 in Supplement 1). The results regarding shift and uptake for government vs freedom framing described here are presented in eTable 4 in Supplement 1.

Among respondents with high vaccine concern, freedom framing, compared with government framing, was associated with a pairwise preference shift of 33.8 percentage points (95% CrI, 21.5-45.8 percentage points; opt-out ignored) toward vaccination and an increased estimate of 6.3 percentage points (95% CrI, 2.9-11.5 percentage points) in estimated overall vaccine uptake for the best vaccine profile. However, the association of framing with uptake varied by the vaccine profile, with the mixed profile showing the highest increase at 15.1 percentage points (95% CrI, 9.1-21.7 percentage points) and the worst profile showing the smallest increase at 0.1 percentage points (95% CrI, 0.03-0.2 percentage points). Among respondents with low vaccine concern, no associations were observed for either pairwise preferences or absolute uptake.

Among respondents with a high VABI, freedom framing, compared with government framing, was associated with a pairwise preference shift of 27.7 percentage points (95% CrI, 14.5-40.5 percentage points; opt-out ignored) toward vaccination and an increase of 4.6 percentage points (95% CrI, 1.8-8.8 percentage points) in estimated overall vaccine uptake for the best vaccine profile. However, the association of framing with overall uptake varied by the vaccine profile, with the mixed profile showing the highest increase at 13.1 percentage points (95% CrI, 6.6-19.9 percentage points) and the worst profile showing the smallest increase at 0.1 percentage points (95% CrI, 0.04-0.3 percentage points). Among respondents with low VABI, no associations were observed for either pairwise preferences or absolute uptake.

In contrast, protect-others framing showed positive preference shifts and uptake compared with government framing. However, interaction terms for protect-others vs government framing were not statistically significant (next section) for vaccine concern or VABI. Consistent with standard practice, simple effects are interpreted as associations rather than subgroup-specific differences. Accordingly, despite similar shifts under higher hesitancy, the absence of moderation indicated that protect-others framing was not selectively associated with acceptance among hesitant respondents.

### Moderation Analyses

We expected that freedom framing would be associated with increased vaccine acceptance among more hesitant respondents, while government framing would show the reverse pattern. We tested this association by examining interaction terms that represent freedom framing vis-à-vis government framing from the hierarchical bayesian models for both vaccine concern and VABI (eTables 2 and 3 in Supplement 1). In the interest of completeness, we also present the interaction terms for protect-others vis-à-vis government framing (eTable 5 in Supplement 1).

Compared with government framing, vaccines described using freedom framing were associated with higher acceptance, and this association was greater for respondents with high vaccine concerns (β = 0.22; 95% CrI, 0.11-0.32) and high VABI (β = 0.16; 95% CrI, 0.05-0.26). In contrast, the parallel interaction for protect-others vs government framing included 0 in the CrIs for both vaccine concerns (β = 0.02; 95% CrI, −0.09 to 0.12) and VABI (β = 0.01; 95% CrI, −0.09 to 0.12). The estimated coefficient of protect-others compared with government framing showed a positive average preference weight (β = 0.15; 95% CrI, 0.04-0.25).

## Discussion

Based on the premise that autonomy concerns partly undergird vaccine hesitancy^9,38^ and that vaccine acceptance is responsive to messaging,^39^ this cross-sectional study examined whether framing vaccines as freedom enhancing would be associated with higher vaccine acceptance compared with the typical government recommendation framing. To this end, we conducted a DCE in which we varied 5 traditional attributes of vaccines, including infection prevention efficacy, severe disease and death protection efficacy, severe adverse effects, minor adverse effects, and duration of protection. The most critical attribute we observed was infection prevention, which aligns with prior research^40^ and suggests that severe disease protection may serve as a meaningful focus when the vaccine does not prevent primary infection, highlighting its greater importance compared with severe adverse effects. The focal attribute was additional reason to take the vaccine, which was varied as government framing, freedom framing, and protect-others framing.

Freedom framing was meaningfully and selectively associated with increases in vaccine acceptance among respondents with pronounced vaccine concerns or adverse beliefs about vaccines, though such increases varied by vaccine profile characteristics, without a negative association with those without such concerns. Likewise, government framing was associated with a noticeable decrease in vaccine acceptance among more hesitant respondents. We also observed that less hesitant respondents generally showed no association with vaccine acceptance by framing. Protect-others framing was associated with increased vaccine acceptance across hesitancy levels. These findings were robust to 2 different operationalizations of vaccine hesitancy: vaccine concern, an attitudinal indicator, and VABI, a belief-based indicator. Together, the findings suggest that the more hesitant respondents may have been responsive to describing the personal freedom benefits of vaccination.

The finding regarding protect-others framing deserves special mention. We included this framing because often, vaccine communication invokes social responsibility as a reason to vaccinate. This reason has little to do with personal freedom; thus, we did not have a specific anticipation about whether and how vaccine hesitancy would modify the association between protect-others framing and vaccine acceptance. We found that protect-others framing was associated with increased vaccine acceptance regardless of hesitancy levels, albeit at a smaller magnitude than freedom framing among respondents who were more hesitant.

### Limitations

Several limitations merit consideration. The sample was older than the average US population,^36^ in part due to the survey item defaulting to age 59 years, and predominantly identified as White race, which may limit generalizability to younger and more diverse populations. The DCE design, while robust for preference elicitation, evaluates hypothetical rather than actual vaccination behavior. Associations with more subtle frame variations remain to be tested. Finally, although freedom framing was associated with consistent shifts in stated preferences, future research should test this association in actual campaigns to assess durability, ecologic validity, and targeted specificity. Data were collected in May 2024 when concerns about COVID-19 were not prominent; thus, the findings should be interpreted in that context. Finally, although this research drew motivation from autonomy-related processes, it did not measure or document any process mechanisms associated with the findings.

## Conclusions

This cross-sectional study found that across 2 independent indicators of vaccine hesitancy—vaccine concerns and vaccine adverse belief endorsement—message framing was differentially associated with stated vaccine acceptance compared with government compliance framing. Freedom framing showed selective associations, with higher acceptance observed among individuals with greater vaccine hesitancy but not among those with lower vaccine hesitancy. In contrast, protect-others framing was associated with positive acceptance overall, with no evidence that these associations varied by hesitancy level. Together, these findings suggest that nonauthority-based message framing may influence stated vaccine acceptance through distinct pathways, with implications for both targeted and broadly applicable communication strategies.

## Notes

Supplement 1. **eAppendix 1.** DCE Survey Development **eAppendix 2.** Quality Control Procedures **eAppendix 3.** Measures and DCE Model **eAppendix 4.** Questionnaire and Respondent Facing Information **eFigure 1.** Sample Screenshot of DCE **eFigure 2.** Average Relative Importance of Attributes **eTable 1.** Regression Results From the Base Model of Vaccine Preferences **eTable 2.** Regression Results From the Model With Moderation of Vaccine Concerns on Vaccine Preferences **eTable 3.** Regression Results From the Model With Moderation of Vaccine Adverse Belief Index on Vaccine Preferences **eTable 4.** Association Between Freedom Framing and Preferences Across Vaccine Profile Scenarios **eTable 5.** Association Between Protect-Others Framing and Preferences Across Vaccine Profile Scenarios
eReferences.

Supplement 2.
Data Sharing Statement
