# Supplementary material for: COVID-19 Vaccine Framing and Acceptance Among Adults Who Are Vaccine Hesitant
Source: JAMA Netw Open. 2026 Mar 31;9(3):e264114. doi: 10.1001/jamanetworkopen.2026.4114 (PMC13040398; doi:10.1001/jamanetworkopen.2026.4114)
Supplement: Supplement 1. — eAppendix 1. DCE Survey Development eAppendix 2. Quality Control Procedures eAppendix 3. Measures and DCE Model eAppendix 4. Questionnaire and Respondent Facing Information eFigure 1. Sample Screenshot of DCE eFigure 2. Average Relative Importance of Attributes eTable 1. Regression Results From the Base Model of Vaccine Preferences eTable 2. Regression Results From the Model With Moderation of Vaccine Concerns on Vaccine Preferences eTable 3. Regression Results From the Model With Moderation of Vaccine Adverse Belief Index on Vaccine Preferences eTable 4. Association Between Freedom Framing and Preferences Across Vaccine Profile Scenarios eTable 5. Association Between Protect-Others Framing and Preferences Across Vaccine Profile Scenarios eReferences. [file jamanetwopen-e264114-s001.pdf]

Supplemental Online Content

Krishnamurthy P, Hu Y. Vaccine framing and acceptance among adults who are vaccine hesitant. *JAMA Netw Open*. 2026;9(3):e264114. doi:10.1001/jamanetworkopen.2026.4114

eAppendix 1. DCE Survey Development

eAppendix 2. Quality Control Procedures

eAppendix 3. Measures and DCE Model

eAppendix 4. Questionnaire and Respondent Facing Information

eFigure 1. Sample Screenshot of DCE

eFigure 2. Average Relative Importance of Attributes

eTable 1. Regression Results From the Base Model of Vaccine Preferences

eTable 2. Regression Results From the Model With Moderation of Vaccine Concerns on Vaccine Preferences

eTable 3. Regression Results From the Model With Moderation of Vaccine Adverse Belief Index on Vaccine Preferences

eTable 4. Association Between Freedom Framing and Preferences Across Vaccine Profile Scenarios

eTable 5. Association Between Protect-Others Framing and Preferences Across Vaccine Profile Scenarios

eReferences

This supplemental material has been provided by the authors to give readers additional information about their work.

© 2026 Krishnamurthy P et al. *JAMA Network Open*.

eAppendix 1. DCE Survey Development

DCE attribute selection was informed by referring to a systematic review of vaccine preference studies using discrete choice experiments[1]. This review synthesized choice-based experiments across vaccine types and identified core attributes frequently examined in vaccine preference research, including effectiveness, safety, and duration of protection.

We selected six attributes based on this review: infection-prevention efficacy, severe-disease protection efficacy, chance of minor side effects, chance of major adverse effects, duration of protection, and additional reason for taking the vaccine (message framing). The distinction between infection prevention efficacy and severe disease protection was motivated by COVID-19 vaccines' differential performance across these outcomes, making this distinction relevant to real-world vaccine decision-making.

Attribute levels were informed by ranges reported in vaccine trials and public health communications. The DCE design and the survey integration (from CloudResearch to Qualtrics to Conjointly and back) was pilot tested to assess comprehension and task feasibility. The pilot data was not formally analyzed. While the pilot did not alter attribute selection, it informed sampling strategy: we added political affiliation as a recruitment criterion to ensure ideological diversity in the final sample and included a political orientation question in the survey to verify balance.

© 2026 Krishnamurthy P et al. *JAMA Network Open*.

eAppendix 2. Quality Control Procedures

Data quality was ensured through multiple mechanisms. The Conjointly platform implemented automated screening for low-quality responses, including: (1) unusually rapid survey completion (e.g., full survey completed within 20 seconds), (2) rapid selection of DCE choice sets without adequate consideration (e.g., selections made within 1 second), (3) absence of mouse movement when a mouse device was detected, and (4) lack of scrolling behavior when required to view full question content. Additionally, two attention checks were used: "I work fourteen months a year" (correct response: No) and "For this question, select 'Slightly unlikely'" (correct response: Slightly unlikely). In cases where respondents completed the DCE part more than once (through a refresh), only the first response that passed both Conjointly quality checks and survey attention checks was included in the analysis.

© 2026 Krishnamurthy P et al. *JAMA Network Open*.

eAppendix 3. Measures and DCE Model

This section focuses on details of the measurement of the primary and secondary second-level factors and extended explanation of the DCE model.

Vaccine concerns

The vaccine concerns measure was based on 12 items below measured on a 1- to 6-point agreement scale ranging from strongly disagree to strongly agree. These are the measures reported in previous research[2]. After reverse coding items marked with an asterisk*, they were averaged to form the vaccine concerns.

1. I feel safe after being vaccinated.*
2. I can rely on vaccines to stop serious infectious diseases.*
3. I feel protected after getting vaccinated.*
4. Although most vaccines appear to be safe, there may be problems that we have not yet discovered.
5. Vaccines can cause unforeseen problems in children.
6. I worry about the unknown effects of vaccines in the future.
7. Vaccines make a lot of money for pharmaceutical companies, but do not do much for regular people.
8. Authorities promote vaccination for financial gain, not for people's health.
9. Vaccination programs are a big con.
10. Natural immunity lasts longer than a vaccination.
11. Natural exposure to viruses and germs gives the safest protection.
12. Being exposed to diseases naturally is safer for the immune system than being exposed through vaccination.

Vaccine Adverse Belief Index (VABI)

The Vaccine Adverse Belief Index was created by computing the arithmetic mean of the responses to the following 10 items assessed on a 6-point agreement scale ranging from strongly disagree (1) to strongly agree (6). These items were mostly drawn from the list of COVID-19 vaccine myths described by Cleveland Clinic and Johns Hopkins Medicine websites[3,4].

1. We can't trust COVID-19 vaccines because they were rushed.
2. The vaccine will give me COVID-19.
3. We don't know what's in these vaccines.
4. These vaccines will alter my DNA.
5. Since COVID-19's survival rate is so high, I don't need a vaccine.
6. The vaccine will cause infertility.
7. I don't think you can trust what the media, medical community, CDC/FDA, or politicians say about the COVID-19 vaccines.
8. Vaccines can cause autism (or other serious health conditions).
9. The vaccine trial involved potentially unethical practices.
10. I think they are trying to insert a microchip inside me as part of the vaccination.

© 2026 Krishnamurthy P et al. *JAMA Network Open*.

Hierarchical Bayes Analysis of the Multinomial Logit Regression

Model overview.
Respondents' choices among Vaccine A, Vaccine B, and a no-vaccine option were modeled as a function of vaccine attributes, including efficacy, side effects, duration of protection, and the additional reason to vaccinate (government compliance, personal freedom, or protect others), along with a no-choice indicator. All vaccine attributes and the no-choice indicator entered the utility function as random coefficients. In second-level models, respondent-level mean preferences were allowed to vary by vaccine hesitancy, operationalized separately using vaccine concerns and the vaccine adverse belief index (VABI), each defined by median splits.

We modeled individual choices in the DCE using a hierarchical Bayesian random effect multinomial logit regression. The probability that individual i chose vaccine alternative j from the tth choice set was

(S1) $$\Pr(y_{it} = j) = \frac{\exp(u_{itj})}{\sum_l \exp(u_{itl})}$$

where l = 1, 2, 3 represents the two alternatives and no-choice in a choice set. Individual i's utility of the choice, u_{itj}, was a linear function of vaccine attributes x_{ijk} $(k = 1, \dots, K)$:

(S2) $$u_{itj} = \sum_k \beta_{ik} x_{ijk}$$

The independent variables x_{ijk} $(k = 1, \dots, K)$ include a dummy coded no-choice and effect-coded vaccine attributes. The specific variables are reported in the results table (eTable S1).

Using the stated choices from the DCE alone, we specified the respondent-level β coefficients as following a multivariate normal distribution with mean vector $\bar{\beta}$ (dimension $k \times 1$) and covariance matrix Σ (dimension $k \times k$):

(S3) $$\beta_{ik} \sim MVN(\bar{\beta}, \Sigma)$$

Furthermore, we estimated the model using the stated choices from the DCE and the vaccine attitude variables based on survey response. Essentially, the respondent-specific β coefficients in Equation S2 were modeled as a linear function of each respondent's survey response variable (e.g., Vaccine concerns, political party affiliations). The distribution specification in 3 became

(S4) $$\beta_{ik} \sim MVN\left(\sum_m \alpha_{mk} z_{im}, \Sigma\right)$$

where z_{im} $(m = 1, \dots, M)$ were respondent i's survey response covariates. From this model, we estimated how the β coefficients of vaccine attributes were linked to Vaccine concerns (eTable S4) or Vaccine Adverse Belief Index (eTable S5). We analyzed the data in SAS® version 9.4, using the procedure PROC BCHOICE. Estimations were conducted using Gamerman Metropolis sampling with 30,000 samples as burn-ins, and the posterior summary was based on 5,000 draws (50,000 draws with thinning of 10). Convergence was reached according to posterior autocorrelations and diagnostic plots.

© 2026 Krishnamurthy P et al. *JAMA Network Open*.

eAppendix 4. Questionnaire and Respondent Facing Information

Following informed consent, the respondents in this study first saw a series of screens followed by a discrete choice experiment. Following this, they responded to a series of questions. The screens prior to the DCE and the questions that followed are presented below.

*****************DCE Screens Begin*******************

Screen 1: Welcome to the Study on Vaccination!
Thank you for participating in this study on vaccination-related attitudes and opinions.
As part of the study, we will show you some hypothetical vaccine choices and ask you to choose.
Keep in mind that these choices are hypothetical. Always consult your doctor or other qualified health care provider with any questions regarding your health.

Screen 2: Vaccine Choice Task
In this part of the study, we will show you several screens. Each screen will describe two COVID-19 vaccines. Please read the descriptions of the vaccines and indicate which vaccine you will choose (or choose neither of them).
The vaccines will be described using the following five features:

- Efficacy in preventing COVID infection
- Efficacy in preventing hospitalization or death if infected with COVID
- Chance of minor side effects of the vaccine
- Chance of major adverse effects of the vaccine
- Durability of protection offered by the vaccine

We will describe each of the features in the next screens.

Screen 3: Efficacy in Preventing COVID Infection
This is a measure of how well vaccination works under real-world conditions to protect people against COVID infection. It is measured between 0% to 100%, where 0% indicates that the vaccination offers no protection, and 100% indicates that the vaccination offers full protection against infection.

Screen 4: Efficacy in Preventing COVID Hospitalization and Death
This is a measure of how well vaccination works under real-world conditions to protect people against hospitalization and death if someone gets infected with the virus. It is measured between 0% to 100%, where 0% indicates that the vaccination offers no protection, and 100% indicates that the vaccination offers full protection against hospitalization and death if someone gets infected.

Screen 5: Chance of Minor Side-effects of the Vaccine
This describes the chance of getting minor side-effects from the vaccine such as short-term fever or body-ache. Such side-effects usually go away in a couple of days and are easily treated with over-the-counter medications such as Tylenol.
Screen 6: Chance of Severe Side-effects

© 2026 Krishnamurthy P et al. *JAMA Network Open*.

This describes the chance that people experience severe side effects that will require hospitalization or long-term treatment. Severe side effects of the vaccine are rare. They can range from 1 in 100,000 to 1 in 1,000,000 vaccinated persons experiencing severe side-effects.

Screen 7: Duration of Protection offered by Vaccine
Vaccines vary in the length of time that they can effectively prevent hospitalization or death if infected. This duration can range from six months to ten years.

Screen 8: Vaccine Features Recap
To recap. We described vaccines using the following five features:

- Efficacy in preventing COVID infection
- Efficacy in preventing hospitalization or death if infected with COVID
- Chance of minor side effects of the vaccine
- Chance of major adverse effects of the vaccine
- Durability of protection offered by the vaccine

We will now describe your task in the next screen.

Screen 9: Vaccine Choice Task
We will now show several screens.
Each screen, will show you two vaccines, A and B, described using the features we just showed you in the previous screen.
Based on the vaccine features, please indicate your preference by clicking "Choose" corresponding to the vaccine. If you do not want either vaccine, you can click on "I will not choose either vaccine".
After every choice, you will see another screen that will have the same layout of choices. However, the details of the vaccines will be different. So, please read the options carefully and indicate your preference.
Depending on your screen size, you may need to scroll right or scroll down to see all of the information before making your selection.

Screen 10-21 (DCE Choices)

Screen 22

Next, we'll ask you a few questions about you, and your views about vaccines in general. Click "Continue" to proceed.

*****************Post DCE Questions Begin*******************

Please indicate your current vaccination status

- I have not taken any of the COVID vaccines
- I have taken one dose of the COVID Vaccine
- I have taken two doses of the COVID vaccine but no boosters
- I have taken two doses of the COVID vaccine and the booster dose(s)

© 2026 Krishnamurthy P et al. *JAMA Network Open*.

Please respond to the following questions (yes/no options presented):

- Do you encourage others around you to get the COVID vaccine?
- Would you take a booster COVID shot against new variants if it becomes available for you?
- I work fourteen months a year. (Attention Check #1)
- Do you typically get the flu vaccine?

Please indicate the extent to which you agree with the following statements about vaccines and vaccinations (assessed on six-point agreement scale, ranging from Strongly Disagree to Strongly Agree):

1. I feel safe after being vaccinated.
2. I can rely on vaccines to stop serious infectious diseases.
3. I feel protected after getting vaccinated.
4. Although most vaccines appear to be safe, there may be problems that we have not yet discovered.
5. Vaccines can cause unforeseen problems in children.
6. I worry about the unknown effects of vaccines in the future.
7. Vaccines make a lot of money for pharmaceutical companies, but do not do much for regular people.
8. Authorities promote vaccination for financial gain, not for people's health.
9. Vaccination programs are a big con.
10. Natural immunity lasts longer than a vaccination.
11. Natural exposure to viruses and germs gives the safest protection.
12. Being exposed to diseases naturally is safer for the immune system than being exposed through vaccination.

Please indicate the extent to which you agree with the following statements about the COVID-19 Vaccines

1. We can't trust COVID-19 vaccines because they were rushed.
2. The vaccine will give me COVID-19.
3. We don't know what's in these vaccines.
4. These vaccines will alter my DNA.
5. I already had COVID-19, so I won't benefit from the vaccine. (Not included in composite score because it is not factually inaccurate)
6. Since COVID-19's survival rate is so high, I don't need a vaccine.
7. Once I get the vaccine, I won't have to wear a mask or worry about social distancing. (Not included in composite score because social distancing and mask wearing was not current at the time of data collection)
8. Now that we have vaccines, the pandemic will be over very soon. (Not included in composite score because pandemic was not in effect at the time of data collection)
9. The vaccine will cause infertility.

Over the last 2 weeks (14 days), how often have you been bothered by the following problems *regarding the Coronavirus Pandemic*?

© 2026 Krishnamurthy P et al. *JAMA Network Open*.

- Feeling nervous, anxious, or on edge
- Not being able to stop or control worrying
- Feeling down, depressed, or hopeless
- Little interest or pleasure in doing things

Please indicate the extent to which you agree with the following statements about the COVID-19 Vaccines

- I don't think you can trust what the media, medical community, CDC/FDA, or politicians say about the COVID vaccines.
- Vaccines can cause autism (or other serious health conditions).
- The vaccine trial involved potentially unethical practices.
- I think they are trying to insert a microchip inside me as part of the vaccination.
- The vaccine is not appropriate for kids. (Not included in composite score because there is debate as to the value of COVID19 vaccine for kids, and would not be factually inaccurate)

Have you been directly impacted by the Coronavirus pandemic?

- Lost Income
- Tested Positive for COVID
- Know a family member that was impacted by COVID
- Worried about financial stability.
- Worried about job stability.
- I have never used a computer.* (Attention Check # 2 not used because of potential confusion with the question.)

Who else lives with you?

- Partner/Spouse
- Kids
- Pets
- Parent(s)
- Siblings/Others

Please indicate your ethnicity?

- White/Anglo
- African American
- Hispanic
- Asian
- Native American/Pacific Islander
- Multi-ethnic
- I do not want to provide my ethnicity
- Other (specify) ____

Please indicate your gender:

- Male
- Female

© 2026 Krishnamurthy P et al. *JAMA Network Open*.

- I do not want to provide my gender.
- Other (Please specify) ____

Please indicate your highest education level:
- Some high school
- Completed high school diploma
- Some undergraduate education
- Completed undergraduate degree
- Completed graduate or professional degree

Please indicate your approximate annual income. The scale is in thousands of dollars (pick 200 if greater than 200,000):

	0	20	40	60	80	100	120	140	160	180	200
Slide to your income level											

My age is (only whole numbers 18 or greater):

	18	26	34	43	51	59	67	75	84	92	100
Age											

Please indicate your political affiliation:
- Republican
- Democrat
- Independent

For this question, select, "Slightly unlikely"*:
- Extremely likely
- Moderately likely
- Slightly likely
- Neither likely nor unlikely
- Slightly unlikely
- Moderately unlikely
- Extremely unlikely

*Attention Check #3.

This concludes the survey. Please click next, and it will take you to the survey platform and display the confirmation message.

© 2026 Krishnamurthy P et al. *JAMA Network Open*.

eFigure 1. Sample Screenshot From the DCE

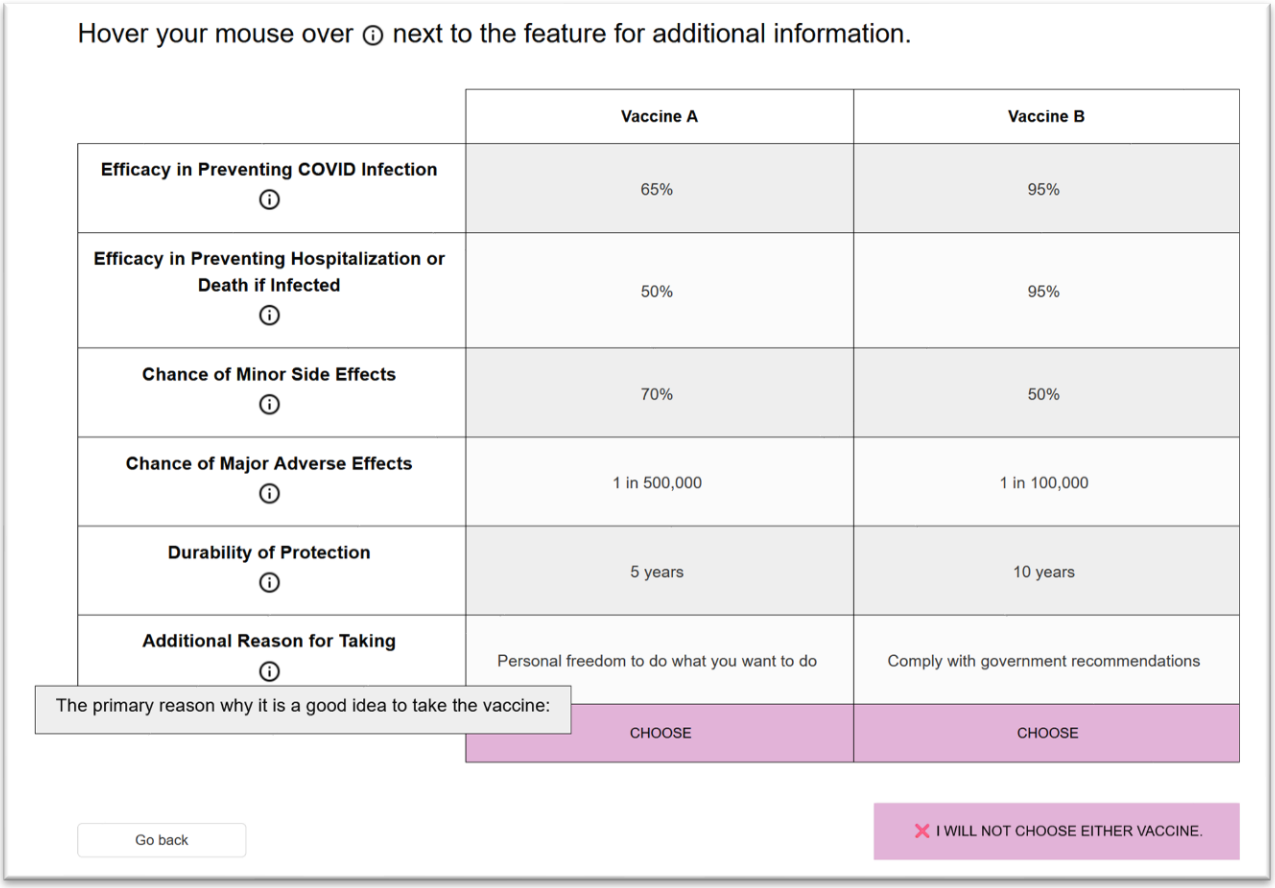
Hover your mouse over ⓘ next to the feature for additional information.

	Vaccine A	Vaccine B
Efficacy in Preventing COVID Infection ⓘ	65%	95%
Efficacy in Preventing Hospitalization or Death if Infected ⓘ	50%	95%
Chance of Minor Side Effects ⓘ	70%	50%
Chance of Major Adverse Effects ⓘ	1 in 500,000	1 in 100,000
Durability of Protection ⓘ	5 years	10 years
Additional Reason for Taking ⓘ	Personal freedom to do what you want to do	Comply with government recommendations
	CHOOSE	CHOOSE

The primary reason why it is a good idea to take the vaccine:

Go back

I WILL NOT CHOOSE EITHER VACCINE.

Note. Each respondent saw 12 screens similar to the illustration above. On each screen, they were asked to pick one vaccine or choose neither. The order of the attributes was fixed. The hover description is shown here to illustrate what the respondents see when they hover the mouse over the attribute in the first column.

© 2026 Krishnamurthy P et al. *JAMA Network Open*.

eFigure 2. Average Relative Importance of Attributes

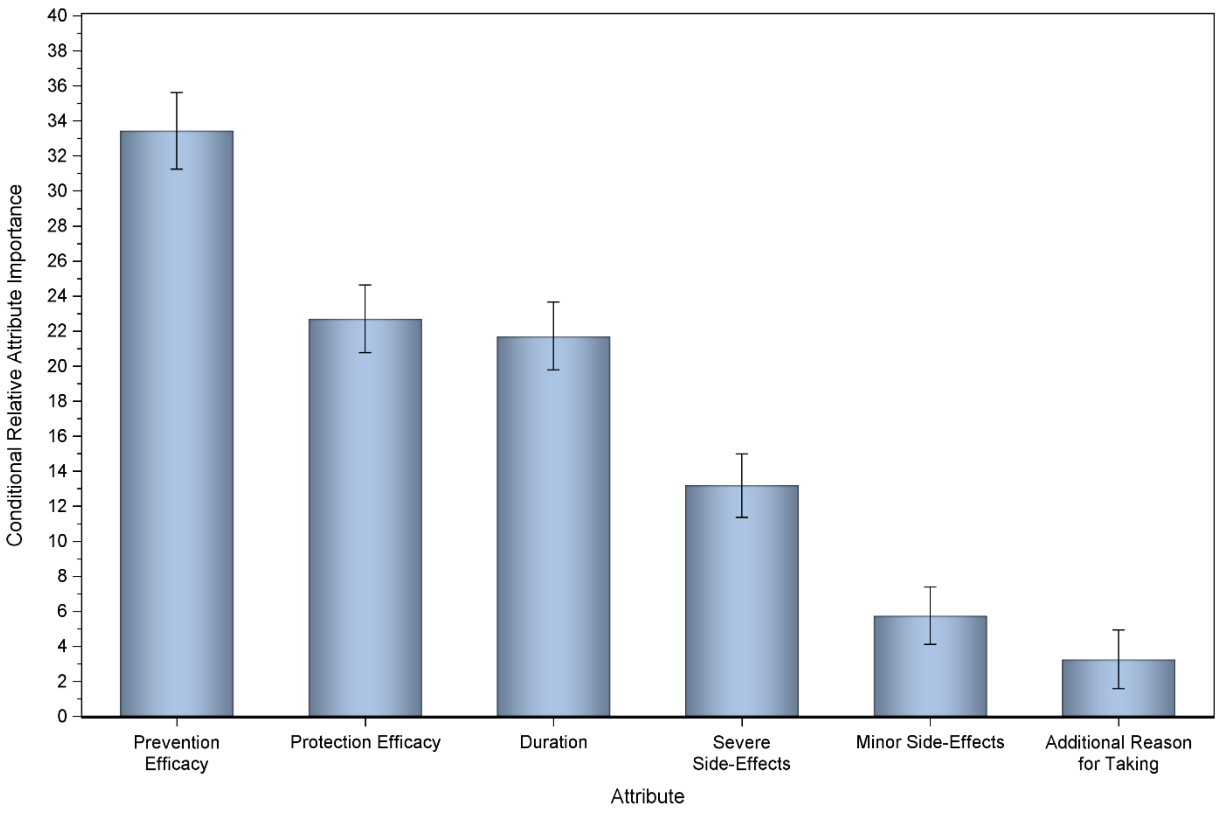

***Note**:* Conditional average importance scores were derived from posterior draws of a Bayesian multinomial logit model, with estimates reported in eTable S1. For each posterior draw and respondent, attribute-level part-worth utilities were recovered from effects-coded estimates by computing the omitted reference level as the negative sum of the estimated levels within each attribute. For each draw, attribute importance was calculated as the range of part-worth utilities within an attribute (maximum minus minimum), divided by the sum of ranges across all attributes for that draw, and scaled to sum to 100. The plotted values represent posterior means across draws, with 95% credible intervals reflecting uncertainty in the posterior distribution.

© 2026 Krishnamurthy P et al. *JAMA Network Open*.

eTable 1. Regression Results From the Base Model of Vaccine Preferences

Attribute	Level	Mean (SD)		95% Credible Interval
Infection Prevention Efficacy	65%	-0.89	0.07	[-1.04,-0.75]
Infection Prevention Efficacy	80%	0.75	0.07	[0.62,0.88]
Infection Prevention Efficacy	95%+	1.85	0.08	[1.69,2.01]
Severe Disease Protection Efficacy	65%	-0.55	0.06	[-0.68,-0.43]
Severe Disease Protection Efficacy	80%	0.6	0.06	[0.47,0.72]
Severe Disease Protection Efficacy	95%+	1.18	0.07	[1.04,1.32]
Minor Side Effects	70%	0.05	0.05	[-0.05,0.14]
Minor Side Effects	90%	-0.33	0.05	[-0.44,-0.23]
Severe Side Effects	1 in 500,000	0.01	0.05	[-0.09,0.11]
Severe Side Effects	1 in 1,000,000	0.7	0.06	[0.58,0.81]
Duration	5 Years	0.44	0.05	[0.34,0.54]
Duration	10 Years	0.93	0.06	[0.81,1.06]
Reason	Freedom	0.05	0.05	[-0.06,0.16]
Reason	Protect Others	0.14	0.05	[0.04,0.25]
No Choice		2.2	0.32	[1.58,2.83]

Note. Coefficients are mean part-worth utilities from an effects-coded discrete-choice model. For each attribute, the omitted level is implicitly defined as the negative sum of the reported levels within that attribute.

© 2026 Krishnamurthy P et al. *JAMA Network Open*.

eTable 2. Regression Results From the Model With Moderation of Vaccine Concerns on Vaccine Preferences

Variable	Mean	(SD)	95% Credible Interval
Infection Prevention Efficacy = 65%	-0.88	0.07	[-1.02,-0.74]
Infection Prevention Efficacy = 80%	0.73	0.07	[0.61,0.86]
Infection Prevention Efficacy = 95%+	1.84	0.08	[1.68,2.01]
Severe Disease Protection Efficacy = 65%	-0.54	0.06	[-0.67,-0.41]
Severe Disease Protection Efficacy = 80%	0.59	0.06	[0.47,0.72]
Severe Disease Protection Efficacy = 95%+	1.18	0.07	[1.03,1.32]
Minor Side Effects = 70%	0.05	0.05	[-0.05,0.15]
Minor Side Effects = 90%	-0.33	0.06	[-0.44,-0.23]
Severe Side Effects = 1 in 500,000	0.02	0.05	[-0.08,0.12]
Severe Side Effects = 1 in 1,000,000	0.7	0.06	[0.58,0.81]
Duration = 5 Years	0.44	0.05	[0.35,0.54]
Duration = 10 Years	0.94	0.06	[0.82,1.06]
Reason = Freedom	0.05	0.05	[-0.05,0.16]
Reason = Protect Others	0.15	0.05	[0.05,0.25]
No Choice	2.31	0.31	[1.69,2.90]
VC × (Infection Prevention Efficacy = 65%)	0.19	0.07	[0.05,0.32]
VC × (Infection Prevention Efficacy = 80%)	-0.14	0.06	[-0.26,-0.01]
VC × (Infection Prevention Efficacy = 95%+)	-0.22	0.07	[-0.37,-0.08]
VC × (Severe Disease Protection Efficacy = 65%)	0.12	0.06	[-0.01,0.24]
VC × (Severe Disease Protection Efficacy = 80%)	-0.08	0.06	[-0.19,0.04]
VC × (Severe Disease Protection Efficacy = 95%+)	-0.27	0.07	[-0.40,-0.13]
VC × (Minor Side Effects = 70%)	-0.04	0.05	[-0.14,0.05]
VC × (Minor Side Effects = 90%)	-0.03	0.05	[-0.13,0.08]
VC × (Severe Side Effects = 1 in 500,000)	0.05	0.05	[-0.05,0.14]
VC × (Severe Side Effects = 1 in 1,000,000)	-0.02	0.05	[-0.12,0.09]
VC × (Duration = 5 Years)	-0.05	0.05	[-0.15,0.04]
VC × (Duration = 10 Years)	0.07	0.06	[-0.04,0.19]
VC × (Reason = Freedom)	0.22	0.05	[0.11,0.32]
VC × (Reason = Protect Others)	0.02	0.05	[-0.09,0.12]
VC × No Choice	2.24	0.29	[1.68,2.82]

Note. VC = Vaccine Concerns. Coefficients are mean part-worth utilities from an effects-coded discrete-choice model. Estimated attribute level coefficients represent average attribute utilities across levels of the moderator; VC moderation terms represent symmetric deviations from this average by VC status.

eTable 3. Regression Results From the Model With Moderation of Vaccine Adverse Belief Index on Vaccine Preferences

Attribute	Mean	(SD)	95% Credible Interval
Infection Prevention Efficacy = 65%	-0.89	0.07	[-1.03,-0.75]
Infection Prevention Efficacy = 80%	0.74	0.07	[0.61,0.87]
Infection Prevention Efficacy = 95%+	1.85	0.08	[1.69,2.00]
Severe Disease Protection Efficacy = 65%	-0.55	0.07	[-0.68,-0.43]
Severe Disease Protection Efficacy = 80%	0.6	0.06	[0.47,0.72]
Severe Disease Protection Efficacy = 95%+	1.18	0.07	[1.04,1.32]
Minor Side Effects = 70%	0.04	0.05	[-0.05,0.14]
Minor Side Effects = 90%	-0.33	0.05	[-0.44,-0.22]
Severe Side Effects = 1 in 500,000	0.02	0.05	[-0.08,0.12]
Severe Side Effects = 1 in 1,000,000	0.69	0.06	[0.58,0.81]
Duration = 5 Years	0.44	0.05	[0.34,0.54]
Duration = 10 Years	0.94	0.06	[0.82,1.05]
Reason = Freedom	0.05	0.06	[-0.05,0.16]
Reason = Protect Others	0.15	0.05	[0.04,0.25]
No Choice	2.22	0.32	[1.61,2.83]
VABI × (Infection Prevention Efficacy = 65%)	0.08	0.07	[-0.05,0.21]
VABI × (Infection Prevention Efficacy = 80%)	-0.14	0.06	[-0.26,-0.01]
VABI × (Infection Prevention Efficacy = 95%+)	-0.28	0.07	[-0.42,-0.13]
VABI × (Severe Disease Protection Efficacy = 65%)	0.12	0.06	[0.00,0.24]
VABI × (Severe Disease Protection Efficacy = 80%)	-0.09	0.06	[-0.20,0.03]
VABI × (Severe Disease Protection Efficacy = 95%+)	-0.29	0.07	[-0.42,-0.15]
VABI × (Minor Side Effects = 70%)	-0.03	0.05	[-0.13,0.06]
VABI × (Minor Side Effects = 90%)	-0.05	0.05	[-0.15,0.05]
VABI × (Severe Side Effects = 1 in 500,000)	-0.02	0.05	[-0.11,0.08]
VABI × (Severe Side Effects = 1 in 1,000,000)	-0.02	0.05	[-0.12,0.09]
VABI × (Duration = 5 Years)	-0.03	0.05	[-0.13,0.07]
VABI × (Duration = 10 Years)	0.05	0.06	[-0.06,0.16]
VABI × (Reason = Freedom)	0.16	0.05	[0.05,0.26]
VABI × (Reason = Protect Others)	0.01	0.05	[-0.09,0.12]
VABI × No Choice	2.02	0.29	[1.45,2.61]

Note. Vaccine Adverse Belief Index = VABI. Coefficients are mean part-worth utilities from an effects-coded multinomial logit model. Estimated attribute level coefficients represent average attribute utilities across levels of the moderator; VABI moderation terms represent symmetric deviations from this average by VABI status.

© 2026 Krishnamurthy P et al. *JAMA Network Open*.

eTable 4. Association Between Freedom Framing and Preferences Across Vaccine Profile Scenarios

Second Level Factor	Group	Preference Shift [95% Credible Interval]	Vaccine Profile	Estimated Uptake Increase [95% Credible Interval]
Vaccine Concerns	High Vaccine Concerns	33.8 [21.5,45.8]	best	6.34 [2.87,11.52]
			medium	3.41 [1.56,6.17]
			mixed	15.09 [9.09,21.66]
			worst	0.10 [0.03,0.22]
	Low Vaccine Concerns	-9.9 [-22.1,2.8]	best	-0.01 [-0.03,0.00]
			medium	-3.71 [-8.60,1.13]
			mixed	-0.44 [-1.14,0.12]
			worst	-0.87 [-2.40,0.22]
VABI	High VABI	27.7 [14.5,40.5]	best	4.60 [1.81,8.76]
			medium	2.88 [1.20,5.41]
			mixed	13.09 [6.64,19.85]
			worst	0.13 [0.04,0.32]
	Low VABI	-4.2 [-16.6,9.0]	best	-0.01 [-0.03,0.01]
			medium	-1.65 [-6.64,3.70]
			mixed	-0.23 [-1.02,0.55]
			worst	-0.26 [-1.24,0.52]

***Note**:* Preference shift is the pairwise percentage-point change in preference for freedom framing ("personal freedom to do what you want to do") versus government-compliance ("comply with government recommendations") framing, excluding opt-out choices. Estimated uptake increase is the percentage-point change in the probability of choosing any vaccine (vs. opting out) when framing shifts from government compliance to freedom framing, holding all other attributes constant. Probabilities are computed from utilities implied by the regression model. Scenarios vary by vaccine profile: best (95% infection prevention; 95% severe disease prevention; 1 in 1,000,000 severe side-effect risk; 90% minor side-effect rate; 10-year duration); worst (50%; 50%; 1 in 100,000; 50%; 6-month duration); medium (65%; 65%; 1 in 500,000; 70%; 5-year duration); mixed (80%; 65%; 1 in 100,000; 90%; 5-year duration). All estimates are based on 5,000 posterior draws from hierarchical Bayesian multinomial logit models.

© 2026 Krishnamurthy P et al. *JAMA Network Open*.

eTable 5. Association Between Protect-Others Framing and Preferences Across Vaccine Profile Scenarios

Second Level Factor	Group	Preference Shift [95% Credible Interval]	Vaccine Profile	Estimated Uptake Increase [95% Credible Interval]
Vaccine Concerns	High Vaccine Concerns	29.1 [16.2,41.7]	best	5.61 [2.30,10.44]
			medium	2.76 [1.13,5.24]
			mixed	12.65 [6.65,19.27]
			worst	0.08 [0.02,0.19]
	Low Vaccine Concerns	5.0 [-7.3,16.9]	best	0.01 [-0.01,0.02]
			medium	1.77 [-2.54,6.21]
			mixed	0.20 [-0.27,0.77]
			worst	0.50 [-0.74,2.00]
VABI	High VABI	25.6 [12.5,38.2]	best	4.31 [1.64,8.31]
			medium	2.61 [1.00,4.99]
			mixed	12.01 [5.69,18.62]
			worst	0.12 [0.03,0.30]
	Low VABI	7.7 [-4.7,20.2]	best	0.01 [-0.01,0.03]
			medium	2.93 [-1.75,8.15]
			mixed	0.39 [-0.25,1.25]
			worst	0.51 [-0.30,1.65]

***Note*:** Preference shift is the pairwise percentage-point change in preference for protect-others ("help prevent spread of disease") versus government-compliance ("comply with government recommendations") framing, excluding opt-out choices. Estimated uptake increase is the percentage-point change in the probability of choosing any vaccine (vs. opting out) when framing shifts from government compliance to protect-others, holding all other attributes constant. Probabilities are computed from utilities implied by the regression model. Scenarios vary by vaccine profile: best (95% infection prevention; 95% severe disease prevention; 1 in 1,000,000 severe side-effect risk; 90% minor side-effect rate; 10-year duration); worst (50%; 50%; 1 in 100,000; 50%; 6-month duration); medium (65%; 65%; 1 in 500,000; 70%; 5-year duration); mixed (80%; 65%; 1 in 100,000; 90%; 5-year duration). All estimates are based on 5,000 posterior draws from hierarchical Bayesian multinomial logit models.

© 2026 Krishnamurthy P et al. *JAMA Network Open*.

eReferences

1. Diks ME, Hiligsmann M, van der Putten IM. Vaccine preferences driving vaccine-decision making of different target groups: a systematic review of choice-based experiments. *BMC Infect Dis*. 2021;21(1):879. doi:10.1186/s12879-021-06398-9

2. Martin LR, Petrie KJ. Understanding the dimensions of anti-vaccination attitudes: The vaccination attitudes examination (VAX) scale. *Annals of Behavioral Medicine*. 2017;51(5):652-660.

3. 9 Common COVID-19 Vaccine Myths Explained – Health Essentials from Cleveland Clinic. Accessed March 29, 2021. https://health.clevelandclinic.org/8-common-covid-19-vaccine-myths-explained/

4. COVID-19 Vaccines: Myth Versus Fact | Johns Hopkins Medicine. Accessed March 29, 2021. https://www.hopkinsmedicine.org/health/conditions-and-diseases/coronavirus/covid-19-vaccines-myth-versus-fact

© 2026 Krishnamurthy P et al. *JAMA Network Open*.
