# Supplementary material for: COVID-19 Vaccine Framing and Acceptance Among Adults Who Are Vaccine Hesitant
Source: JAMA Netw Open. 2026 Mar 31;9(3):e264114. doi: 10.1001/jamanetworkopen.2026.4114 (PMC13040398; doi:10.1001/jamanetworkopen.2026.4114)
Supplement: Supplement 2. — Data Sharing Statement [file jamanetwopen-e264114-s002.pdf]

Data Sharing Statement

Data
Data available: Yes
Data types: Deidentified participant data
How to access data: The deidentified participant data and accompanying data dictionary that support the findings of this study will be made available from the corresponding author upon reasonable request, including by the journal's review team or following publication.
When available: With publication

Supporting Documents
Document types: None

Additional Information
Who can access the data: The deidentified participant data and data dictionary will be made available to qualified researchers upon reasonable request to the corresponding author, including to the journal's review team or following publication.
Types of analyses: The data may be used to verify the reproducibility of the reported findings. Researchers wishing to extend or build upon the findings are encouraged to contact the corresponding author to discuss potential collaboration or coordinated use of the data.
Mechanisms of data availability: The data will be made available with investigator support after approval of a reasonable request and, when appropriate, completion of a simple data use agreement to ensure proper acknowledgment and responsible use.
